# Morning Salivary Cortisone Versus Serum Cortisol in the Overnight Dexamethasone Suppression Test (ODNST): Evaluation in a Clinical Setting

**DOI:** 10.1111/cen.15233

**Published:** 2025-03-21

**Authors:** Mathilde Mordaunt, Adrian Heald, Waseem Majeed, Rupinder Kochhar, Akheel Syed, Rajshekhar N. Mudaliar, Ramadan Alshames, Fahmy Hanna, David Marshall, Brian Keevil, Anthony A. Fryer

**Affiliations:** ^1^ University of Manchester Manchester UK; ^2^ Department of Endocrinology and Diabetes Salford Royal Hospital Salford UK; ^3^ Biochemistry Department, Faculty of Dentistry Tripoli University Tripoli Libya; ^4^ Department of Endocrinology and Diabetes University Hospital North Midlands Stoke on‐Trent UK; ^5^ Department of Biochemistry Manchester University NHS Trust Manchester UK; ^6^ Keele University Keele UK

**Keywords:** adrenal insufficiency, overnight dexamethasone suppfession test, salivary cortisone, sensitivity, specificity

## Abstract

**Introduction:**

Saliva hormone measurement is increasingly being applied in everyday clinical practice. In relation to salivary cortisone measurement, there is a particular advantage, with minimal chance of cross‐reaction with prescribed glucocorticoids and greater convenience. We evaluated the utility of salivary cortisone measurement in patients undergoing an overnight dexamethasone suppression test (ONDST).

**Methods:**

Individuals undergoing an ONDST had parallel measurement of serum cortisol and salivary cortisone at 0900 following midnight dexamethasone (1 mg). Salivary cortisone was measured by electrospray positive liquid chromatography tandem mass spectrometry. The threshold for adequate suppression of salivary cortisone was< 2.7nmol/L; serum cortisol was< 50nmol/L.

**Results:**

Results for 34 individuals which included 21% men (mean age 49.4 years) and 79% women (mean age 56.7 years) were analysed. Serum cortisol did not suppress in 22/34 cases. Salivary cortisone did not suppress in two of the cases where cortisol did suppress. We found a strong correlation between 0900 salivary cortisone and serum cortisol after 1 mg ONDST (*r* ^2^ = 0.65, *p* = 0.009). When performance of post‐dexamethasone salivary cortisone (< 2.7nmol/L) alone in relation to suppression of serum cortisol (< 50nmol/L) was analysed all but 2 individuals were correctly classified. They had values for post dexamethasone salivary cortisone/serum cortisol of respectively 5.9/23 nmol/L (presented with unexplained fatigue, case 25) and 7/32 nmol/L (investigated for cyclical Cushing's Syndrome that was excluded, case 29). Agreement was 94.1%, kappa 0.87, *p* < 0.0001. The sensitivity of salivary cortisone for potential Cushing's syndrome as indicated by the post‐dexamethasone 0900 serum cortisol was 100% (all cases of potential cortisol excess (0900 cortisol > 50nmol/L) were picked up) and specificity of 84.6% with a positive predictive value of 90.5% of salivary cortisone (using serum cortisol as the standard) and negative predictive value of 100% in relation to ruling out cortisol excess.

**Conclusion:**

We have provided further evidence that ONDST salivary cortisone has potential to be the first‐line test for suspected Cushing's syndrome, not requiring venepuncture or attendance at hospital, with 100% sensitivity and reasonable specificity. Application of the salivary cortisone test has the potential for significant savings of money and time in this and other contexts.

## 1 Introduction

The overnight dexamethasone suppression test (ONDST) is a well‐recognised test, that is, used to screen for cortisol excess (Cushing's syndrome). If there is failure to suppress cortisol, further investigation is required to confirm the diagnosis [1]. However, there is no agreement on the gold standard test that should be used to screen for Cushing's syndrome [2, 3].

Recent developments in mass spectrometry have meant that it is possible accurately to measure steroids such as cortisone in the saliva, specifically using liquid chromatography tandem mass spectrometry [4]. There is increasing evidence to suggest that salivary cortisone levels can be determined to spare the patient coming into hospital for an ONDST [1].

In the salivary gland, corticosteroid 11‐beta‐dehydrogenase isozyme 2 converts cortisol into cortisone which is inactive [5]. Cortisone is the favoured steroid for salivary measurements over cortisol because cortisol is naturally found at lower levels in the saliva. Furthermore, it has been shown to have a more linear correlation to total serum cortisol [1]. It is also relevant that studies have shown that salivary cortisol can be affected by confounding factors such as topical preparations containing cortisol. However, salivary cortisone levels are not affected by such factors [1, 4].

There are limitations of the ONDST. For example the majority of cortisol is bound in the blood to cortisol binding globulin hormone (CBG) and to albumin [6, 7]. The measurement of total serum cortisol is thus dependent on the level of binding proteins in an individual, which can vary for multiple reasons between individuals, including the taking of estrogen containing preparations, pregnancy, and sepsis. Furthermore individual differences in dexamethasone metabolism cannot be accounted for without parallel measurement of serum dexamethasone levels which is rarely done in eery day clinical practice [8] Non‐bound serum cortisol would be a superior measure of circulating cortisol but this is offered by only a limited amount of laboratories, as it is much more labour intensive and complicated [9]. An ONDST requires the patient to attend hospital or an alternative clinical setting for the blood test [1, 8]. Additionally, the act of drawing blood can trigger cortisol release due to stress adding another potential interference [9] This less relevant in relation to morning than late evening blood tests. Provision of a salivary sample does not entail either of these issues.

We report the application of salivary cortisone measurements in comparison with serum cortisol measurements in the outpatient setting in individuals undergoing an overnight dexamethasone suppression test (ONDST), using 1 mg dexamethasone taken orally at midnight with 0900 cortisol and salivary cortisone measurement on the following day.

## 2 Methods

We undertook a service evaluation of salivary cortisone vs serum cortisol in the ONSDT. Patients undergoing an ONDST had parallel measurement of serum cortisol and salivary cortisone at 0900, following dexamethasone 1 mg at midnight.

All individuals had been seen in the endocrinology clinic at a single centre and either had an adrenal adenoma or were deemed to have features suggestive of Cushing's Syndrome.

Salivary cortisone was measured by electrospray positive liquid chromatography tandem mass spectrometry [10, 11]. Serum cortisol was measured on the Siemens Atellica autoanalyzer (Manchester, UK). The threshold for adequate suppression of salivary cortisone was < 2.7nmol/L, based on the validation reported by Issa et al. in 2023 [1]; serum cortisol was < 50nmol/L. Between‐batch imprecision for cortisol showed coefficient variations of 13.4% to 2.7%across a range of concentrations from 4.2 to 118 nmol/L. Between‐batch imprecision for salivary cortisone showed coefficient variations of 8.6% to 2.3% across a range of concentrations from 5.0 to 130.9 nmol/L. Recovery was 93% and 96% for cortisol and cortisone, respectively. 20‐alpha and 20‐beta dihydrocortisone showed baseline separation with cortisone and did not interfere in the assay.

A higher cut off was also applied of salivary cortisone < 7.5 nmol/L, which correlates with < 138 nmol/L serum cortisol in relation to mild autonomous cortisol secretion (MACS) vs Cushing's syndrome [12].

Ethics permission was not required as this was a service evaluation of a new laboratory technique in comparison with existing practice [13].

### 2.1 Statistical Analysis

Comparison was made between 0900 salivary cortisone and 0900 serum cortisol postmidnight dexamethasone with 0900 cortisol as the standard.

All analyses were conducted using STATA version 16, StataCorp, Texas, USA.

## 3 Results

Results for 34 individuals; 21% men (mean age 49.4 years and 79% women (mean age 56.7 years were analysed. In 59% of individuals an adrenal adenoma was present; in 29% Cushing's Syndrome was suspected on the basis of clinical features

Of the 34 consecutive cases (Table 1) 4 were ultimately found to have adrenal Cushing's and one pituitary Cushing's disease. 17 were adrenal nodules of which 15 showed evidence of MACS. All individuals diagnosed to have adrenal Cushings had a confirmed adrenal adenoma with no evidence of pituitary abnormality on magnetic resonance imaging (MRI) pituitary scan. Biochemistry in each case confirmed adrenal Cushings.

**Table 1:** Individual cases.

Study number	Age (years)	Sex	Body mass index (BMI) (kg/m^2^) at time of ONDST	Reason for the ONDST/endocrine diagnosis	Other diagnoses – major co morbidities –
1	16	Female	32.8	Weight gain and lump on back of neck	None
2	44	Female	28.4	Adrenal Cushing's	Avascular necrosis of the right hip Osteoporosis Metatarsal fractures
3	78	Female	22.0	Adrenal nodule (MACS)	Bladder cancer Past malignant melanoma and basal cell carcinoma Hypertension Lichenoid keratosis Asthma
4	52	Female	26.0	Hypertension	Hypertension Hypercholesterolemia Right thyroid sub centric U2 nodules Mixed anxiety and depression disorder
5	80	Female	36.1	Adrenal Cushing's	Depression Hypokalaemia of uncertain origin
6	68	Female	38.7	Adrenal Cushing's	OSA Diabetes mellitus type 2 Hyperlipidaemia Obesity
7	57	Female	23.2	Adrenal adenoma (MACS)	Hypertension
8	28	Male	26.4	Hypertension	CKD stage G4A3 Familial hypertension Smoker
9	45	Female	24.0	Adrenal Cushing's	Hypertension Monoclonal IgG ‐gammopathy
10	92	Female	28.3	Adrenal nodule (MACS)	Osteoarthritis Osteoporosis
11	57	Female	44.6	Weight gain	Obesity Previous gastric bypass surgery Mixed anxiety and depressive disorder Degenerative joint disease of the hand Hypothyroidism
12	41	Female	28.0	Fatigue and weight gain	Chronic fatigue Mixed anxiety and depression IBS Irregular menses Axillary hyperhidrosis
13	57	Female	23.2	Adrenal adenoma (MACS)	Hypertension
14	66	Female	30.1	Adrenal adenoma (MACS)	Emphysema
15	60	Male	28.0	Adrenal adenoma (MACS)	Diabetes mellitus type 2 Emphysema
16	54	Female	33.2	Non‐functioning pituitary microadenoma	Anxiety Bilateral sensorineural hearing deficit
17	28	Female	29.6	Unexpected weight gain	Nil
18	68	Female	35.1	Adrenal adenoma (MACS)	Roux‐en‐Y bypass (2009) Knee replacement Hypertension Asthma GORD Recurrent depressive disorder
19	57	Female	23.2	Adrenal adenoma (MACS)	Hypertension
20	76	Male	40.3	Bilateral adrenal adenomata (MACS)	Left lower lobe thyroid nodule Hypertension Diabetes PE (2022) BPH TIA Osteoarthritis Laminectomy
21	60	Female	Not available	Adrenal adenoma (MACS)	Anxiety and depression Constipation Hypothyroidism Total abdominal hysterectomy and oophorectomy
22	88	Female	40.2	Adrenal adenoma (MACS)	Hypertension Right knee osteoarthritis Complete heart block – pacemaker
23	28	Male	31.0	Stress fracture with no obvious trauma	Vitamin D deficiency Foot stress fractures
24	61	Female	28.5	Bilateral adrenal adenomata (MACS)	Hypercholesterolaemia Hypertension Lumbar spine fracture in past
25	42	Female	28.8	Unexplained fatigue and weight gain	Fibromyalgia Chronic fatigue Osteoarthritis
26	35	Female	29.8	Unexplained weight gain	Anxiety
27	70	Male	24.1	Adrenal adenoma	Hypertension Impaired glucose tolerance Recurrent UTIs Occupational lung disease GORD
28	72	Female	37.3	Bilateral adrenal adenomata (MACS)	Ischaemic heart disease Hypertension Hyperlipidaemia Iron deficiency anaemia – has infusions of iron
29	48	Male	36.1	Query cyclical Cushing's syndrome, ultimately excluded	MS Asthma Non‐alcoholic fatty liver disease Hyperlipidaemia Erectile dysfunction
30	72	Female	31.3	Bilateral adrenal adenomata with right adrenalectomy but persistently high cortisol post operatively – Likely Adrenal MACS	Hypertension Previous cholecystectomy and incisional hernia repair Hypothyroidism Smoker Asymmetric tonsil under ENT
31	39	Female	37.1	Adrenal adenoma (MACS)	Obesity Diabetes mellitus type 2 Hypertension Bilateral renal stones
32	36	Male	48.5	Unexplained weight gain, already under endocrine follow‐up for silent pituitary corticotroph adenoma that was resected (2011)	Obesity Pituitary macroadenoma resected 2011
33	28	Female	34.4	Cushing's Disease	Pituitary microadenoma – resected Hypertension Anxiety Obesity
34	73	Female	31.1	Adrenal adenoma	Gallstones Hypertension Hypothyroidism Coeliac disease Osteoarthritis

Serum cortisol failed to suppress < 50nmol/L in 22/34 cases. Salivary cortisone did not suppress in two of the cases where cortisol did suppress to < 50nmol/L.

We found a strong correlation between 0900 salivary cortisone and serum cortisol after 1 mg ONDST (*r* ^2^ = 0.65, *p* = 0.009)

**Figure 1:**
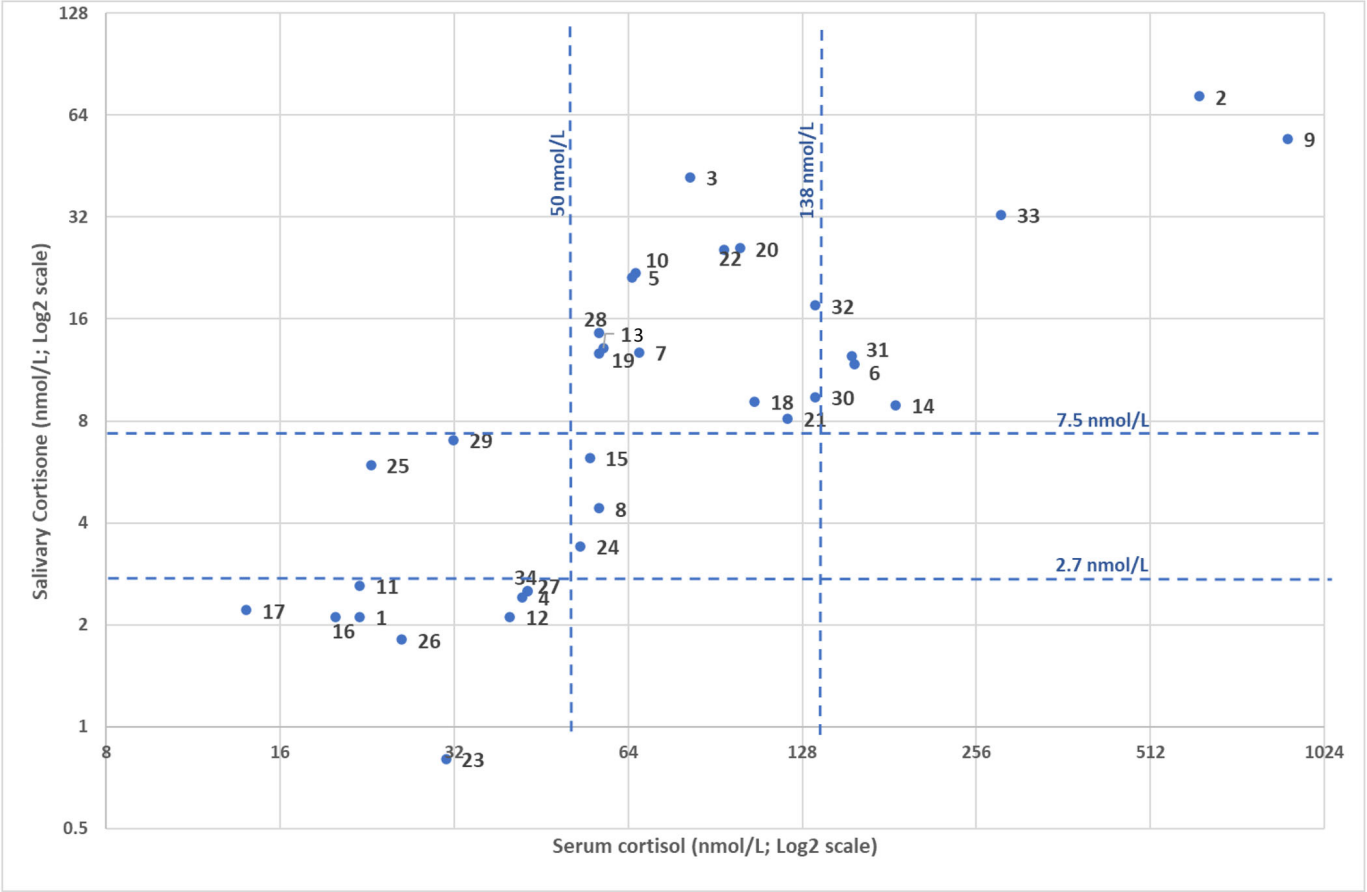
Post‐ midnight dexamethasone 0900 serum cortisol (log scale) vs salivary cortisone (log scale) for 34 consecutive individuals. The lower and higher cut points for salivary cortisone (2.7 and 7.5 nmol/L – horizontal dashed lines) and serum cortisol (50 and 138 nmol/L – vertical dashed lines) relate respectively to the cut points for the ONDST as a screening test for any cortisol excess and separately for MACS vs other causes of cortisol excess. Saliva and blood samples were taken at the same time.

When performance of post‐dexamethasone salivary cortisone (< 2.7 nmol/L) alone in relation to suppression of serum cortisol (< 50 nmol/L) was analysed (Figure 1) all but two were correctly classified. These had above threshold salivary cortisone with below threshold cortisol with values for salivary cortisone/serum cortisol of 5.9/23 nmol/L (presented with unexplained fatigue, case 25) and 7/32 nmol/L (investigated for cyclical Cushing's Syndrome that was excluded, case 29) post dexamethasone.

The sensitivity of salivary cortisone for Cushing's syndrome as indicated by the post‐dexamethasone 0900 serum cortisol was 100%, that is, all cases of cortisol excess were picked up and specificity of 84.6% with a positive predictive value of 90.5% of salivary cortisone (using serum cortisol as the standard) and negative predictive value of 100% in relation to ruling out cortisol excess (Figure 1). Agreement was 94.1%, kappa 0.87, *p* < 0.0001.

Using a salivary cortisone cut‐off of 7.5 nmol/L to include MACS, of the three who had a 0900 post‐Dexamethasone cortisol of between 50 and 137 nmol/L, the values of salivary cortisone/serum cortisol respectively were, 3.4/53 nmol/L (bilateral adrenal incidentalomata, case 24); 6.2/55 nmol/L (adrenal adenoma, case 15) and 4.4/57 nmol/L (hypertension, case 8). Sensitivity of the salivary cortisone in relation to serum cortisol for the higher cut‐off for salivary cortisone remained 100%. Thus no cases of potential cortisol excess were missed.

## 4 Discussion

The diagnosis of Cushing's syndrome is one of the most challenging in endocrinology. We have demonstrated test validity and potential clinical utility of salivary cortisone in the ONDST as the first line test. This supports the finding of previous studies in this area [1, 4, 9, 14]. This study also showed some overlap between serum cortisol and salivary cortisone around the individual cut off values, but this is not surprising because it is increasingly recognised that cortisol excess is a continuous variable and dichotomous cut offs may not be appropriate. Importantly, in our study no cases of potential cortisol excess that would have been identified by an ONDST were missed (no false negatives) and based on a salivary cortisone threshold of 2.7 nmol/l only two cases were misidentified by postmidnight dexamethasone salivary cortisone, when compared with the equivalent serum cortisol (false positive). Taking the MACS higher cut point for serum cortisol and salivary cortisone three individuals (2/3 with an adrenal adenoma) of the individuals fell into the new reference range, but again no people with a 0900 ONDST > 137 mmol/L were missed using salivary cortisone as the first line screening test.

Although in this case the saliva sample for cortisone estimation was taken in the outpatient department, there is no reason why this sample could not be taken at home and then dropped off at the hospital laboratory specimen reception or posted to the hospital laboratory. Postage of saliva samples is allowed through the regular mail in the UK as in many other countries [9].

Regarding the two cases where the salivary cortisone level was above the threshold for adequate suppression, this is not surprising as there is not absolute concordance between results; in the paper by Issa et al. (ref) there were nine false positives with a serum cortisol < 50nmol/L and saliva cortisone> 2.7nmol/L. It is almost inevitable that discrepancies between serum cortisol and salivary cortisone after midnight dexamethasone are noted because they are being compared with each other rather than with another independent gold standard test.

Salivary cortisone could therefore be used as an alternative sampling method which does not require venepuncture or attendance at hospital. Application of the saliva test has the potential for significant savings of money and time and a frameshift in the paradigm of screening for Cushing's syndrome. Furthermore there is increasing evidence for the value of salivary cortisone in the investigation of potential adrenal insufficiency [15].

Our findings are supportive of studies that have looked at various groups in relation to application of salivary sampling. Notably Ng et al. [16] looked at the potential use of late‐night salivary cortisone and the ONDST in screening for Cushing's syndrome. Salivary cortisone was found to be superior in terms of its relation to total serum cortisol and more accurate at diagnosing inadequate cortisol suppression than salivary cortisol.

In a 2005 study by Viardot et al. [17], night time salivary cortisol was compared to 24 h urinary free cortisol and the ONDST. Healthy volunteers (*n* = 20), patients with Cushing's syndrome (*n* = 12), obese patients (*n* = 16), pregnant women (*n* = 20) and some patients who have suspected Cushing's syndrome (*n* = 14) were included. The authors found that night‐time salivary cortisol was reproducible in healthy volunteers with low day to day variability and that it appeared to be a preferable alternative to 24 h urinary free cortisol as a first‐line screening test for Cushing's syndrome.

A further study by Elias et al., showed that late night salivary cortisol was able to diagnose cortisol excess despite a normal 24 h urinary free cortisol in 17.3% of Cushing's syndrome and thus had superior diagnostic performance [18]. This is supported by a meta‐analysis published by Carroll et al. in 2009 with total of 947 patients (339 with Cushing syndrome) across nine studies [19]. The authors recommended late night salivary cortisol as a robust and convenient test for screening and diagnosis of Cushing's syndrome. However a major drawback for salivary cortisol measurement vs salivary cortisone is potential contamination from topical preparations containing cortisol. These preparations can markedly increase cortisol concentration in saliva samples whereas cortisone concentration is not affected [20]. It should also be stated that there is a confounding effect of shift work on late night cortisol or cortisone measurements [17].

Regarding screening for Cushing's syndrome in people with adrenal incidentalomas, a recent retrospective study was carried using 173 patients. These patients had an ONDST done and diurnal measurements of saliva cortisol/cortisone with samples being collected at 21:00, the middle of the night and 9:00am after also measuring the dexamethasone levels. Results indicated a strong relation between salivary cortisone and serum cortisol following 1 mg of dexamethasone (r = 0.95) [1]. In addition, a similar retrospective study done by Berndt et al., used 209 patients (155 with adrenal incidentalomas, 54 with possible Cushing's syndrome). These patients had two saliva samples taken (2300 and then 0800 post ONDST [13]. The results showed that 8 am salivary cortisone and late night salivary cortisone were more sensitive than late night salivary cortisol as a screening method for people with Cushing's syndrome [14], further supporting the use of salivary cortisone over salivary cortisol.

Efthymiadis et al. [2] published the first study to compare late night salivary cortisol, late‐night salivary cortisone, urinary free cortisol, ONDST and to ascertain which had the best diagnostic capability for identifying Cushing's syndrome over Cushing's disease or mild autonomous cortisol secretion. In a retrospective single centre study they reported that late night salivary cortisol, late‐night salivary cortisone and ONDST individually offer comparable performance, superior to urinary free cortisol in screening for hypercortisolism. ONDST was deemed preferable for the identification of mild autonomous cortisol secretion (MACS). The authors proposed a simple screening algorithm based on late night salivary cortisone in combination with ONDST for screening moderate/high clinical pre‐test probability patients, and ONDST for adrenal incidentalomas [2].

Regarding the diagnosis of MACS, the revised European Society for Endocrinology 2023 guideline recommends testing adrenocorticotropic hormone (ACTH)‐independency by demonstrating suppressed or low‐normal morning plasma ACTH levels and a repeat DST to confirm MACS [21]. Although the updated guideline emphasizes the confirmation of ACTH‐independency, the diagnosis of MACS still relies on the 1 mg ONDST.

In relation to limitations, we accept that the number of cases reported here is relatively low. Furthermore, serum or salivary dexamethasone level was not evaluated in all individuals. However, all cases were consecutive and we have been able to provide relevant clinical details on all of them. This was a pragmatic evaluation in a real world clinical setting.

We accept that, at present, only a limited number of laboratories can offer the LCMS salivary cortisone assay. If this approach is seen as viable, there will be enough evidence to enable the LCMS analysis to be available more widely in the United Kingdom and elsewhere.

Updated guidelines recommend the use of at least two screening tests when investigating Cushing's syndrome and there is intermediate to high pre‐test probability, because the diagnostic accuracy increases significantly [22]. The authors of the paper recommended the combination of late night salivary cortisone and ONDST as the gold standard for diagnosis of autonomous cortisol secretion. Combining the two tests in an outpatient setting would allow the patient to collect both saliva samples at home and post them to the laboratory.

It is clear from this service evaluation and the published research that the use of salivary cortisone as an assay for screening and diagnosing Cushing's syndrome, using liquid chromatography tandem mass spectrometry, has huge potential for saving money, saving the patient a trip to hospital and mitigating the need for venepuncture as the first line test in screening for Cushing's Syndrome.

In conclusion, Cushing's syndrome is hard to diagnose as it has a variety of presentations, sometimes slowly manifesting over a number of years, obesity being one of them. There needs to be a low threshold for screening for cortisol excess which implies that a convenient and relatively inexpensive test should be the first‐ line of investigation with a high degree of sensitivity, whether applied as a simple alternative to the ONDST 0900 blood test or with a paired with a late night salivary cortisone on the previous evening, as recommended in a recent review [22].

## Author Contributions

M.M. and A.H.H. wrote the manuscript with data analysis by A.F., W.M., R.K., R.A., A.S., and A.F. contributed to and have approved the final version of the manuscript. B.K., R.M., and F.H. provided senior review.

## Conflicts of Interest

All authors declare: no support from any organisation for the submitted work; no financial relationships with any organisations that might have an interest in the submitted work in the previous 3 years; no other relationships or activities that could appear to have influenced the submitted work.

## Data Availability

The data that supports the findings of the study are available on reasonable request.
